# Supplementary material for: Knowledge and perceptions of snakes, snakebites and their management among health care workers in Sudan
Source: PLoS One. 2024 Sep 20;19(9):e0302698. doi: 10.1371/journal.pone.0302698 (PMC11414984; doi:10.1371/journal.pone.0302698)
Supplement: S1 File — (DOCX) [file pone.0302698.s001.docx]

**Appendix 1**: **Knowledge and perceptions of snakes, snakebites and their management among health workers in Sudan**.

**Invitation letter and consent to participate**

The current study aims to undertake the knowledge, attitude and practices of snakes, snakebites and their management among HCWs. We would appreciate if you give us five minutes of your precious time to help us achieve the study goals.

**Section A**:

| **Sociodemographic Characteristics** |  | | | | | | | | | |
| --- | --- | --- | --- | --- | --- | --- | --- | --- | --- | --- |
| Age in Years |  | | | | | | | | | |
| Gender | Male | | | | Female | | | | | |
| Occupation | Medical doctor | | House officer | | Registrar | Consultant | | Pharmacist | | Nurse |
| Number of years practice | <5 years | | | 5- 10 years | | | | | >10 years | |
| Sector | Private | | | | | | Govermental | | | |
| Previously attended a training | Yes | | | | | | No | | | |
| Areas from which participants acquired skills on snake bite management: | Self-education on the internet or in text books | Learning from senior colleagues or othe health professionals on the job | | | Knowledge and skills gotten during my training in School | | Not applicable | | | |

**Section B**: **Knowlegde About Snakes**

| S.N. | **Knowlegde About Snakes** | **Yes** | **No** | **I don’t know** | | | | | |  |
| --- | --- | --- | --- | --- | --- | --- | --- | --- | --- | --- |
|  | **Knowledge about snakes (Q1 to Q8)** | | | | | | | | |  |
| Q1 | All snakes are poisonous |  |  |  | | | | | |  |
| Q2 | What type of animals are snakes? | Mammals | Birds | Reptiles | Not sure | | | | |  |
| Q3 | All snakes are carnivorous (feed on other animals)? |  |  |  | | | | | |  |
| Q4 | All snakes are venomous, that is, inject “toxins” (venom) into a person after a bite? |  |  |  | | | | | |  |
| Q5 | All snakes have fangs in front of their mouth |  |  |  | | | | | |  |
| Q6 | Snakes pick sounds using their ears? |  |  |  | | | | | |  |
| Q7 | Snakes are important for farmers |  |  |  | | | | | |  |
| Q8 | Deforestation and urbanization has increased human-snake interaction |  |  |  | | | | | |  |
|  | **Knowledge On Snake Bite (Q9- Q17)** | | | | | | | | |  |
| Q9 | Handling a dead snake’s head is safe enough |  |  |  | | | | | |  |
| Q10 | Fang arks can always be seen or found on the victim after every snake bite? |  |  |  | | | | | |  |
| Q11 | Can a person report at the hospital with symptoms of snake bite toxin injection without actually being bitten by a snake after he or she might have been pricked by an object he or she suspected to be a snake? |  |  |  | | | | | |  |
| Q12 | Sleeping under mosquito nets can prevent snakebites |  |  |  | | | | | |  |
| Q13 | Do you think every time a venomous (“poisonous”) snake bites, it always injects venom (poison) into the victim? |  |  |  | | | | | |  |
| Q14 | Signs and symptoms of snake bites are determined by the type of snake responsible for the bite. |  |  |  | | | | | |  |
| Q15 | Which of the following is best used to determine if a person bitten by a snake had venom actually being injected into him or her by the snake? | The type of snake | Signs and symptoms | Presenting complaint from the victim of being bitten by snake | | | | I cant tell | |  |
| Q16 | The signs and symptoms of snake bite depends on the amount of venom injected by the snake. |  |  |  | | | | | |  |
| Q17 | What time of the day do you think snake bites are most common? | During the day | During night | At dawn | | | Not sure | | |  |
|  | **Practice domain (Q18 to Q25)** | | | | | | | | | |
| Q18 | Tourniquet prevents spread of poison in the remaining part of the body |  |  |  | |  | | |  | |
| Q19 | Reassurance should be practice as 1st protocol against prevention |  |  |  | |  | | |  | |
| Q20 | Incision at the site of bite helps removing poison |  |  |  | |  | | |  | |
| Q21 | If bite site is leg , elevating it will reduce poison spread |  |  |  | |  | | |  | |
| Q22 | Bringing snake to treating physician increase chances of survival by correct identification |  |  |  | |  | | |  | |
|  | **Attitude** | | | | | | | | | |
| Q23 | Residing in cities is a protective factor from snake bite |  |  |  | |  | | |  | |
| Q24 | Snake bite is outcome of revenge inspired from past incidents |  |  |  | |  | | |  | |
| Q25 | I can ask my teachers questions and receive a quick response e-learning: |  |  |  | |  | | |  | |

**Section B**:

**Cont. Table 1** The survey domains and individual statements

| S.N. | **Management of snakebites** | | **Very confident** | | **Confident** | **Fairly confident** | **Low confident** | |
| --- | --- | --- | --- | --- | --- | --- | --- | --- |
|  | **Management of snakebites (Q1 to Q7)** | | | | | | | |
| Q1 | How confident are you in the management of snake bites? | |  | |  |  |  |  |
| Q2 | Have you ever managed, supervised or nursed a snake bite patient before? | | **Yes** | | | **No** | | |
| Q3 | On average how often do people report to your health facility with snake bites? | |  | |  |  |  |  |
| Q4 | How many snake bites have you managed or helped or provided some services in its management since the beginning of 2021? | |  | |  |  |  |  |
| Q5 | Does your facility have a protocol for the management of snakebite? | |  | |  |  |  |  |
| Q6 | Do you think your health facility has what it takes to effectively manage snake bites? | |  | |  |  |  |  |
| Q7 | What is/are lacking in the management of snakebite if you think there is inadequacy at your hospital? (State “Not applicable” if your answer to question 41 above is yes or I don’t know) | |  | |  |  |  |  |
|  | **Knowledge regarding management of snakebites (Q1 to Q8)** | | | | | | | |
| Q1 | How do you triage (prioritize) someone with snake bite in your facility? | Emergency | | Urgent | | Not urgent | I don’t know | |
| Q2 | What do you do when people report to your health facility with snakebite? | Refer immediately | | Give first aid treatments and refer | | Admit and treat | Call for assistance from another health facility | |
| Q3 | Which of the following tests will you first recommend when someone reports snakebite to determine there was actually an injection of venom into the person (envenoming)? | Full blood count | | Grouping and cross matching | | 20 minutes whole blood count test (20MWBCT) | | |
| Q4 | Antivenoms are the only specific antidotes in the management of snake bites by venomous snakes. | Yes | | No | | I don’t know | | |
| Q5 | Antivenoms should be given to all patients bitten by snakes? | Yes | | No | | I don’t know | | |
| Q6 | When an anti snake venom is injected into the muscle (intramuscular), it is as effective as when injected into the veins (intravenous) | Yes | | No | | I don’t know | | |
| Q7 | Antisnake venoms remain useful  for months or even years after stated expiry dates | Yes | | No | | I don’t know | | |
| Q8 | In the use of anti snake venoms, it is better to give low doses repeated over several days than give high initial doses. | Yes | | No | | I don’t know | | |
