# Supplementary material for: Knowledge and perceptions of snakes, snakebites and their management among health care workers in Sudan
Source: PLoS One. 2024 Sep 20;19(9):e0302698. doi: 10.1371/journal.pone.0302698 (PMC11414984; doi:10.1371/journal.pone.0302698)
Supplement: Attachment — Submitted filename: R1 Manuscript KAP snakes (1).docx [file pone.0302698.s003.docx]

**Knowledge, Attitude, and Practices of Snakes, Snakebites and their Management among Health Workers in Sudan: Need for Internet-mediated Knowledge Management System for Continuing Professional Development**

Ali Awadallah Saeed^1, 2^, Ahmed Hassan Fahal^2^, Omer Abdelhamid Gibreel^3^, Ayman B. Mousa^1^, Saeed M Omer ^4^, Abdallateif Alkhair Omer ^5^, Intisar A. M. A. Elalawy ^6^

1 Department of Pharmacology and therapeutics, Faculty of Clinical and Industrial Pharmacy, National University-Sudan, E-mail: [aliawadsaeed@nu.edu.sd](mailto:aliawadsaeed@nu.edu.sd) , ORCID: https://orcid.org/0000-0003-3524-4825, Scopus ID: <https://www.scopus.com/authid/detail.uri?authorId=58106128900>

2 Mycetoma Research Center, University of Khartoum

3 Department of Management Information System, College of Business Administration, Gulf University for Science and Technology – [Gibreel.o@gust.edu.kw](mailto:Gibreel.o@gust.edu.kw)

4 Faculty of Medicine and Health Science, Gadarif University, Sudan, [drsaeedomar@yahoo.com](mailto:drsaeedomar@yahoo.com)

5 Faculty of Medicine, Omdurman Islamic University, [abdallateif.1983@gmail.com](mailto:abdallateif.1983@gmail.com)

6 Alrayan College of Health Sciences and Nursing, Pharmacy program, Almadina, Saudi Arabia, ORCID: 0009-0002-1777-1058, E-mail: [intisarelalawy@gmail.com](mailto:intisarelalawy@gmail.com)

**Corresponding author: Ali Awadallah Saeed**

[**aliawadsaeed@nu.edu.sd**](mailto:aliawadsaeed@nu.edu.sd)

**Abstract**

**Background:**

Snakebite is considered one of the world’s most serious medical problems. The WHO recently reconsidered snakebite envenomation as a neglected tropical disease, and called for new strategies to combat it.

**Material and methods**

This descriptive cross-sectional survey was conducted in August 2022. It included 394 medical and healthcare providers from different parts of Sudan. The questionnaire contained seven different sections, including important and basic knowledge of snakes, snake bites and their management. The data collected were entered into Microsoft Excel and analyzed in the Statistical Package for Social Sciences (SPSS) version 20 (IBM SPSSInc., Chicago, IL).

**Results**

A total of 394 medical and health care providers participated. Of theses, there were 44.7% males, and 53.3% females. Only 25.9% of the participants had training in snakebite management, 58.1% answered correctly snake knowledge questions 45.3% answered the snakebite knowledge questions correctly, and 60.4% answered snakebite practice questions correctly. Only 14% had rated themselves as very confident in the management of snakebites, 40.9% stated their health facility has a protocol for managing snakebites.

**Conclusion:**

Intensive educational effort should be focused on basic knowledge of snakes, snakebites, prevention, management of snakebites and first aid measures. With the advent of the internet and tools such as learning management systems, introducing and disseminating knowledge has become easier and faster. Hence, we proposed an internet-mediated Knowledge Management System for Continuing Professional Development.

**Introduction**

Snakebite is considered one of the world’s most serious medical problems, and the WHO recently declared snakebite envenomation a neglected tropical disease (9) and called for new strategies to combat it and resources to implement them (10). There are 5.4 million poisonous snake bites globally, fatal in around 2.5 million bites. Snake bites are accountable for 1,25,000 deaths annually. ^(1)^ WHO reported that 81-95% of snake bites occur in the tropical regions of South Asia, South-East Asia, Sub-Saharan Africa and Latin America. ^(2)^ In sub-Saharan Africa, the number of persons treated in health centers for snakebite envenomation is estimated at 315,000 cases per year, with more than 9,000 amputations and 7,000 deaths. Since many victims do not seek medical care and they are invisible to official reports thus, the actual statistics of snake envenomation remain underestimated. (8).

In some parts of the African continent, the snakebites' burden is higher than that of trypanosomiasis, leishmaniasis, and onchocerciasis, and its mortality can be higher than malaria. The snakebites' high mortality in Africa is multifactorial, including lack of antitoxins, inadequate health care system, and scarcity of rapid access to medical centers.^3^ Furthermore, many inhabitants of rural areas believe in traditional healers and do not seek treatment due to low socioeconomic and health education levels and victims died shortly after the snakebite accident, and that is not reported in the hospital records. In rural regions, where most snakebite occur (8), the general population's awareness of appropriate behavior in response to snakebites is poor. A study in Nigeria showed that most patients had attempted at least one potentially deleterious first aid measure prior to presentation to hospitals. ^(11)^ In Ghana, an intervention to improve snakebite medical care ability and adherence to protocols led to a significant improvement in care and decreased significantly the snakebite mortality post the intervention. ^(12)^

In Sudan, snakebite statistics are lacking, despite the high estimated burden of the problem. ^6^ During the last 70 years, only a few data were reported on the Sudanese venomous snakes. One study reported the presence of 17 medically significant snakes belonging to three major families: Burrowing asps, Elapidae, and Viperidae. These snakes usually become abundant during and after the rainy season, and most snakebite victims are farm workers.^5^ The records of the Natural History Museum, University of Khartoum, since the early last century, revealed some interesting data on snakes in Sudan. ^5,7^

To determine the poor outcome of snakebite management in Sudan, the present study was set out to determine the medical and health care providers' knowledge, attitude and practice of snakebites management. Furthermore, the study proposed that need to introduce an internet based learning management system (LMS) to support the dissemination of knowledge and practices on snakes bite management. There is proposal to use of internet based learning management system to increase awareness about snakebites due to the success of this form of internet-enabled learning in other part of Africa (23-28). For example, South Africa used internet-mediated learning management system to tackle the issue of AIDS (24), In Libera an internet-based learning management system was introduced after the Ebola virus epidemic (23).

**Material and methods**

This descriptive cross-sectional survey was conducted in August 2022. It included 394 medical and health care providers from different parts of Sudan. A validated web-based self-administrated questionnaire was used to collect the required data. The questionnaire included important and basic knowledge of snakes, snake bites and their management. A pilot study was conducted prior to the study to validate the questionnaire. The questionnaire was distributed widely online to medical and health care providers. They gave informed consent.

Sudan Snake Bite Research Network prepared this questionnaire. It contains seven different sections: section one comprised of seven socio-demographic questions on the study participants, section two had eight questions on the participants' knowledge of snakes, section three contained nine questions on knowledge of snakebites, section four contained five questions regarding practice towards snakebites, section five contained two questions regarding attitude toward snakebites, section six had seven questions regarding snakebites management and section seven included ten questions on snakebites management knowledge.

**Data analysis**

The data were entered into Microsoft Excel and managed by the Statistical Package for Social Sciences (SPSS) version 20 (IBM SPSS Inc., Chicago, IL). Frequency and percentage were calculated for qualitative variables, and median and interquartile range (IQR) were calculated for quantitative variables. A total score of knowledge, attitude, and practice was calculated and later classified into good and poor based on the average score. Attitudes to dealing with snake bites were expressed in%. Chi-square was performed to find relationships between good knowledge, practice, and other selected variables. Questions were analyzed to determine the knowledge and practice levels. Each correct answer was given a score of 1, and the "don't know", and "wrong" answers were given 0 points. The result is expressed as%. The results were rated good and bad based on the median.

**Ethical considerations**

Informed oral consent from all study subjects was obtained, and confidentiality was maintained

**Results**

The study included 394 medical and health care providers, of them, there were 176 (44.7%) males, and 218 (53.3%) females, 142 (36%) were less than 40 years old, and 252(64%) aged were more than that. The study participants were pharmacists 122 (31%), medical officers 94 (23.9%), registrars 79 (20%), house officers 46 (11.7%) nurse 27 (6.8%) and consultant 26(6.6%). The study showed that 255 participants (64.7%) had less than five years of medical and health experience, 89 (22.3%) had experience ranging between five and ten years, and 50 (13%) had more than ten years’ experience. Only 102 participants (25.9%) had training in snake bite management, gained from senior colleagues (37%), 28.5% were self-educated, and 10% gained it during training. (Table 1).

There were eight questions on snakes knowledge, and the results showed that 321 (81.5) corrected answered the fact that not all snakes are poisonous, 129 (32.7) stated all snakes are carnivorous, 255 (64.7) said not all snakes are venomous, 113 (28.7) recognized all snakes has fangs in front of their mouth, 213 (54.1) thought snakes pick sounds using their ears, 213 (54.1%) believe snakes are important for farmers and 255 (64.7%) believed deforestation and urbanization have increased human-snake interaction. (Table 2) The study showed that 322 participants (81.7) knew snakes are reptiles. (Table 3) The study showed that 229 participants (58.1%) answered correctly snake knowledge questions while 165 (41.9%) had incorrect answers.

Regarding snakebites' knowledge, there were nine questions. 85 (21.6%) of the participants responded correctly to the question on handling a dead snake's head is not safe enough, 195 (49.5%) recognized the fact that marks can always be seen or found on the victim after every snake bite, 219 (55.6%) believed that a person reported at the hospital with symptoms of snakebite can be given antitoxin injection without actually being bitten by a snake, 122 (31%) believed sleeping under mosquito nets can prevent snakebites. A venomous ("poisonous") snakes always inject venom (poison) into the victim; 151 (38.3%) do not believe this statement, 316 (80.2%) recognized that the snake type determines the symptoms and signs of snakebites and 279 (70.8%) thought that the symptoms and signs of the snakebite depend on the amount of venom injected by the snake. (Table 2)

At least 209 (53%) believed the symptoms and signs could determine the snake and venom injected, and 31 (7.9%) felt the snakebite common occurs during the day. (Table 3) For the snakebites knowledge, 179 participants (45.3%) answered the snakebite knowledge questions correctly, while 215 (54.7%) responded incorrectly.

The obtained data showed that 247 participants (26.7%) believed that a tourniquet prevents the spread of poison in the remaining part of the body, and 320 (81.2%) stated reassurance of the victim should be practice as 1st protocol for prevention. Regarding the incision at the bite site helps remove that poison, that was the belief of 186 participants (47.2%), and 282 (71.6%) thought if the leg is the bite site, its elevation will not reduce poison spread. Bringing the snake to the treating physician increases the chances of survival by correct identification of the snake believed by 297 participants (75.4%). (Table 4)

In this study, 238 participants (60.4%) answered snakebite practice questions correctly, while 156 (39.6%) did not.

Regarding snakebites' attitude, residing in cities is a protective factor against snakebites was stated by 297 participants (75.4%), and 106 (26.9%) said that the snakebite is the outcome of revenge inspired by past incidents. (Table 5)

The study showed only 55 participants (14%) had rated themselves as very confident in the management of snakebites, and 203 participants (51.5%) had previously managed, supervised or nursed a snakebite patient. Regarding management and help provided by participants in snakebite management in 2021, 20 (5.1%) managed more than 100 patients, and 56 (14.2%) managed between 10 to 50 patients. 161 (40.9%) stated their health facility has a protocol for managing snakebites. Only 179 participants (45.4%) said their health facility has what it takes to effectively manage snake bites. (Table 6)

Regarding the management of snakebites, 265 participants (67.3) considered it an emergency. Most of the participants, 178 (45.2%) manage the snakebite by admission and treatment rather than referring patients immediately 26 (6.6%). Sixty-one patients (15.5%) correctly said a 20-minute whole blood count test is the most appropriate test for snakebites management. Antivenoms are the only antidotes in managing snakebites by venomous snakes, as stated by 267 participants (75.4%).

Antivenoms made anywhere in the world are good for the management of snake bite victims, was said by 199 participants (50.5%), and 191 (48.4) believed that antivenoms should be given to all patients bitten by snakes. Fifty-seven participants (14.5%) thought that the intramuscular injection of an anti-snake venom is effective as an intravenous one. One hundred ninety-seven participants (50%) did not know how long the anti-snake venoms could remain useful after the expiry date. In using anti-snake venoms, it is better to give low doses repeated over several days than high initial doses 132 participants (33.6%) believed that is the correct regimen.

**Discussion:**

In a study conducted by Subedi et al among medical students 50.7% were males and 49.3% females (13), in study conducted by Amritesh Kumar was similar 14, 52% males and 48% females, in study conducted by Ali et al 58% were males and 42% were females (21), were as in our study were 44.7% males, and 53.3% females.

In this study 37% participants get their knowledge and skill in snake bite management from their senior colleges, 28.5% self-educated and 24.3% don’t know the skills.

In Kumar and et al study (14), the knowledge domain less than half i.e. (48%) of respondents had good knowledge, which is nearby to our findings 58.1% answered correctly.

100% of the study participants knew that all snakes are not poisonous as stated by Kashif Ali and et al (21). In study conducted by Kumar A and et al, majority of respondents i.e. 88 % correctly responded that all snakes are not poisonous (14), which similar to our study 81.5% respondent not all snakes are not poisonous.

In the Kumar A and et al study (14), less than half (40%) had knowledge that snake bites were fatal, which is less than our findings, more than half (64.7%) said not all snakes are venomous.

More than half i.e. (55%) agreed about the fact that snakes were important for farmers in Kumar A and et al study (14), and this is similar to our findings, 54.1% believe snakes are important for farmers, while 94% of the study participants by Kashif Ali and et al believed that snakes are helpful to farmers (21).

In Kumar a and et al study (14), majority (82%) responded correctly that deforestation and urbanization had increased human-snake interaction, which is high compared to our findings, 64.7% believed deforestation and urbanization have increased human-snake interaction.

In Kumar and et al study (14), tow third (66%) stated correctly that snakes has fangs in front of their mouth, which high compared to our findings 28.7%.

In area regarding snakebites knowledge, 45.3% participants answered the snakebite knowledge questions correctly, while 54.7% responded incorrectly.

Only 21.6% participants stated handling a dead snake's head is not safe enough, which consistent to WHO guidelines, do not handle the snake with your bare hands as even a severed head can bite. (17)

A venomous ("poisonous") snakes always inject venom (poison) into the victim; 38.3% do not believe this statement, 80.2% recognized that the snake type determines the symptoms and signs of snakebites and 70.8% thought that the symptoms and signs of the snakebite depend on the amount of venom injected by the snake. 53% believed the symptoms and signs could determine the snake and venom injected.

Only 7.9% felt the snakebite common occurs during the day, compared to (92%) participants believed that snakes bite mostly during monsoons and at night (21).

In Kumar and et al study (14), in snakebites practice domain and after answering practice questions, only 35 % of respondent had good practice, this low compared to that found in our study, more than half (60.4%) answered snakebite practice questions correctly.

Only 26.7% in this study believed that a tight band (tourniquet) prevents the spread of poison in the remaining part of the body, when compared to study conducted in Srilanka by Anjana et al (15), application of tight band (tourniquet) proximal to the site of bite was considered as an important first aid measure among 74.9% of cases where as it was higher (96%) in a study conducted by Kumar et al. (16) and (89%) in Kumar A et al study (14), compared to study by Kashif Ali, all the study participants said they would tie a tourniquet at the site and would rush to the nearest health facility (21)

In practice domain 89% of inters responded correctly in Kumar and et study (14) that more than half of interns i.e. (59%) responded that practice of reassurance as the first protocol in snake bite management, which is low compared to our findings 81.2% stated reassurance of the victim should be practice as first protocol for prevention.

Bringing snakes to physician in Kumar and et al study (14) , helped in correct identification of species and better patient management were recommended by more than half i.e. 54% which is low compared to our study, three quarter (75.4%). Killed snake should be taken to the dispensary or hospital with the patient in case it can be identified as stated by WHO (17).

The incision at the bite site helps remove that poison correctly answered by 47.2%, which is high and douple to that found by Kumar and et al study (14), 24%. In our findings, 28.4% from respondent correctly answered elevation of leg will reduce poison spread, which is low compared to 62% found by Kumar and et al study. (14)

In attitude domain regarding snakebites attitude, Kumar and et al (14) found almost more than 8% of the interns stated right attitude, that snake bites were outcome of revenge inspired from past incidents and residing in cities is a protective factor from snake bite, which is very low compared to our findings, residing in cities is a protective factor against snakebites was stated by 75.4%, and 26.9% said that the snakebite is the outcome of revenge inspired by past incidents.

In management of snakebites, the study showed only 14% participants had rated themselves as very confident in the management of snakebites, and 51.5% participants had previously managed, supervised or nursed a snakebite patient. Regarding management and help provided by participants in snakebite management in 2021, 5.1% managed more than 100 patients, and 14.2% managed between 10 to 50 patients.

40.9% stated their health facility has a protocol for managing snakebites. Only 45.4% participants said their health facility has what it takes to effectively manage snake bites.

Regarding the management of snakebites, 67.3% participants considered it an emergency. Most of the participants, 178 (45.2%) manage the snakebite by admission and treatment rather than referring patients immediately (6.6%). The patient must be transported to a place where they can receive medical care (dispensary or hospital) as quickly, but as safely and comfortably, as possible as stated by WHO (17).

As stated by WHO guidelines for the management of snakebites (17), 20-minute whole blood clotting test (20WBCT) is most common and very useful and informative laboratory test, in our study only sixty-one patients (15.5%) correctly said a 20-minute whole blood count test is the most appropriate test for snakebites management.

In our study 75.4% participants antivenom is the only specific antidote to snake venom, this consistent with WHO guidelines (17).

50.5% participants stated antivenoms made anywhere in the world are good for the management of snake bite victims. Medical treatment is available for snakebite as stated by all participants and 86% participant knew about availability of treatment for snakebite at PHC study conducted by Kashif Ali and et al (21)

Nearly half of participants (48.4%) believed that antivenoms should be given to all patients bitten by snakes, while in WHO guidelines, antivenom should be given only to patients in whom its benefits are considered likely to exceed its risks, since antivenom is relatively costly and often in limited supply, it should not be used indiscriminately. The risk of reactions should always be taken into consideration [level of evidence E]. (17)

Only 14.5% thought that the intramuscular injection of an anti-snake venom is effective as an intravenous one which is consistent to WHO guidelines, intramuscular blood levels of antivenom never reach those achieved rapidly by intravenous administration (17).

Half of participants (50%) did not know how long the anti-snake venoms could remain useful after the expiry date. Anti-snake venoms can be expected to retain useful activity for months or even years after these dates if stored properly (17). Expired antivenoms could provide an alternative option for snakebite treatment (22).

In using anti-snake venoms, it is better to give low doses repeated over several days than high initial doses, 33.6% believed that is the correct regimen which is not consistent to practice. The recommended dose is often the amount of antivenom required to neutralize the average venom yield when captive snakes are milked of their venom, in practice, the choice of an initial dose of antivenom is usually empirical as stated by WHO (17).

**Clinical implication of the study:**

In Sudan surveillance of snakebites is essential to understand their epidemiology better and reporting of cases must be improved to provide accurate data and to facilitate stock management, training implementation, and appropriate interventions. Now that snakebites are recognized as a Neglected Tropical Disease, a global, adapted, and sustainable approach is required. Training of health care personnel represents a vital aspect of this global approach.

**Technological Implication of the Study:**

Due to the introduction of mobile systems in Africa and the recent development of undersea fiber optics cables, the barrier to accessing the internet-mediated knowledge management system has been dramatically reduced. The development of an internet-mediated knowledge management system that provides a repository of lessons learned and best practices with a combination of video lectures and online material (19, 20) is best way to increase knowledge regarding snakes, snakebites and their Management among health workers in Sudan.

**Strengths and weakness of our study:**

The main strength of the current study is the sample size of healthcare workers participated in the study from areas affected by snakebites (394). In addition, we have conducted a multicenter study in six hospitals from both the public and private sectors. While the limitation include, the lack of long-term evaluation of the knowledge, and exact evaluation of healthcare facilities that recipe patients affected by snakebites.

**Conclusion**

There is a strong need to develop an internet-mediated knowledge management system that would help in Sudan's continuing professional development process in different areas especially in snake, snakebites and management. Recent studies have shown the effectiveness and success of the learning management system (LMS) in Sudan (18).

The present study shown a gap in the knowledge on the snake, snakebite and its management, which should be addressed in different levels, personal, community and at governmental level. This area should be an important part of medical curriculum for all health professionals because they are exposed to such cases in practice.

Poor training in snake bite management, snake and snakebite knowledge, snakebite practice and lack of health facility protocols for management of snakebites management are major concerns.

**Future prospects:**

In order to address these issues we suggest that an intensive educational effort should be focused on basic knowledge of snakes, snakebites, prevention, management of snakebites and first aid measures.

**Conflict of interest**: None

**Source of funding**: Self-funded

**References:**

1. Ch(1)ippaux JP. Snake bites: appraisal of the global situation. Bull world health organ. 1998;(5):515-24.
2. Kasturiratne A, Wickremasinghe AR, de Silva N, Gunawardena NK, Pathmeswaran A, Premaratna R, et al. The global burdedn of snake bite: a literature analysis and modeling based on regional estimates of envenoming and deaths. PloS Med. 2008; 5:218.
3. Huda Khalid, Rasha S Azrag, Retrospective hospital-based study on snakebite envenomation in Sudan, Transactions of The Royal Society of Tropical Medicine and Hygiene, Volume 115, Issue 9, September 2021, Pages 992–997, <https://doi.org/10.1093/trstmh/trab085>
4. Warrell DA. Snake Bite. lancet. 2010; 375:77-88.
5. Kirk R., Poisonous snakes of the Sudan, Sudan Wild life and sport, 1951, vol. II: 17- 27.
6. Kasturiratne A, Wickremasinghe AR, de Silva N et al., The global burden of snakebite: a literature analysis and modeling based on regional estimates of envenoming and deaths, PLoS Med 2008; 5:e218.
7. Corkill NL., A guide for the species represented in the collection of natural history museum Khartoum. Natural History Museum Publication no. 3, 1935.
8. Chippaux JP. Estimate of the burden of snakebites in sub-Saharan Africa: a meta-analytic approach. Toxicon. 2011; 57(4):586–99. https://doi.org/10.1016/j.toxicon.2010.12.022 PMID: 21223975.
9. Organisation WH. Available at <http://www.who.int/neglected> diseases/diseases/en/. Accessed on December 2017. Neglected tropical diseases.
10. Chippaux JP. Snakebite envenomation turns again into a neglected tropical disease! J Venom Anim Toxins Incl Trop Dis. 2017; 23:38. https://doi.org/10.1186/s40409-017-0127-6 PMID: 28804495; PubMed Central PMCID: PMCPMC5549382.
11. Habib AG. Public health aspects of snakebite care in West Africa: perspectives from Nigeria. J Venom Anim Toxins Incl Trop Dis. 2013; 19(1):27. https://doi.org/10.1186/1678-9199-19-27 PMID: 24134780; PubMed Central PMCID: PMCPMC3831819.
12. Visser LE, Kyei-Faried S, Belcher DW. Protocol and monitoring to improve snake bite outcomes in rural Ghana. Trans R Soc Trop Med Hyg. 2004; 98(5):278–83. https://doi.org/10.1016/S0035-9203(03) 00065-8 PMID: 15109550.
13. Subedi N, Paudel IS, Khadka A, Shrestha U, Mallik VB, Ankur KC. Knowledge of first aid methods and attitude about snake bite among medical students: a cross sectional observational study. J Occup Med Toxicol. 2018;13:26.
14. Kumar A, Gopakumar S, Sathyan S, Singh SB, Bhasy BI. A KAP study on snakebite and its management among medical interns in a tertiary care medical college and hospital in Trivandrum district, Kerala. Int J Community Med Public Health 2019;6:1340-4
15. Silva A, Marikar F, Murugananthan A, Agampodi S. Awareness and perception on prevention, first aid and treatment of snake bite among Srilankan farmers. J Occ Med Toxicol. 2014;9:20.
16. Kumar A, Dasgupta A, Biswas D, Sahoo SKS, Preeti PS. Knowledge regarding snake bite in snake bite in rural Bengal-Are they still lingering on myths and misconceptions? IAIM. 2015;2(7);36-41.
17. David A Warrell, Guidelines for the management of snake-bites, WHO Library Cataloguing-in-Publication data, ISBN 978-92-9022-377-4.
18. Gibreel, O. A., Mahdi, M. A., Saeed, A. A. (2021): E-learning and the Digital Divide Role in Transforming Higher Education in Sudan: An Exploratory Case Study Analysis, Vol 9, No. 1/2, pp. 95 -108.
19. Ahmed, A. and Gibreel, O. (2021), "The role of social media in strengthening civic engagement in the Middle East and North Africa", World Journal of Entrepreneurship, Management and Sustainable Development, Vol. 17 No. 3, pp. 309-317. <https://doi.org/10.1108/WJEMSD-02-2021-0020>
20. Gibreel, Omer; Jeon, Seongmin; and Yoo, Byungjoon, "An Intercountry Analysis on the Factors and Barriers to Internet Accessibility", (2013). PACIS 2013 Proceedings. 6. <http://aisel.aisnet.org/pacis2013/6>
21. Kashif Ali, Ishan Pathak, Knowledge, Attitude and Practice regarding Snakes and Snakebite among Interns, Indian Journal of Forensic and Community Medicine, October-December 2017;4(4):229-231.
22. Robert A. Harrison, José María Gutiérrez, The Neutralization Efficacy Of Expired Polyvalent Antivenoms: An Alternative Option, Toxicon. 2019 October; 168: 32–39. doi:10.1016/j.toxicon.2019.06.216
23. Walsh S, et al. Introducing an E-learning Solution for Medical Education in Liberia, Annals of Global Health. 2018; 84(1), pp. 190–197. DOI: <https://doi.org/10.29024/aogh.21>
24. Reisach U, Weilemann M. Organisational aspects and benchmarking of e-learning initiatives: a case study with South African community health workers. Glob Health Promot. 2016 Jun;23(2):57-66. doi: 10.1177/1757975914567178. Epub 2015 Mar 2. PMID: 25733133.
25. Barteit S, Jahn A, Banda SS, Bärnighausen T, Bowa A, Chileshe G, Guzek D, Jorge MM, Lüders S, Malunga G, Neuhann F. E-Learning for Medical Education in Sub-Saharan Africa and Low-Resource Settings: Viewpoint. J Med Internet Res. 2019 Jan 9;21(1):e12449. doi: 10.2196/12449. PMID: 30626565; PMCID: PMC6329426.
26. JK Chime, P Munyati , M Katepa-Bwalya , M Musumali , B Mweetwa , S Kagulura , JS Kachimba, B Vwalika , J Mufunda, Using E-Learning for Skills Transfer, Motivation and Retention of Health Workers in Zambia, Medical Journal of Zambia, Vol. 43 (2): PP 103 -105 (2016).
27. Frehywot, S., Vovides, Y., Talib, Z. et al. E-learning in medical education in resource constrained low- and middle-income countries. Hum Resour Health 11, 4 (2013). <https://doi.org/10.1186/1478-4491-11-4>
28. Rohwer A, Motaze NV, Rehfuess E, Young T. E-learning of evidence-based health care (EBHC) in healthcare professionals: a system atic review. Cam pbell Systematic Reviews 20 17:4, DOI: 10 .40 73/ csr.20 17.4

**Tables:**

**Knowledge, Attitude and Practices of Snakes, Snakebites and their Management among Health Workers in Sudan: Need for internet-mediated** **Knowledge Management System for Continuing Professional Development.**

**Table 1. Socio-demographic characteristics of the study population (n=394)**

| **Variables** | **Character** | **No. (%)** |
| --- | --- | --- |
| Sex | Male | 176 (44.7) |
|  | Female | 218 (53.3) |
| Age | < 40 | 142 (36) |
|  | ≥ 40 | 252 (64) |
| Age | 29.3 ± 8 |  |
| Occupation | Medical officer | 94 (23.9) |
|  | House officer | 46 (11.7) |
|  | Registrar | 79 (20) |
|  | Consultant | 26 (6.6) |
|  | Pharmacist | 122 (31) |
|  | Nurse | 27 (6.8) |
| Working Sector | Governmental | 229 (58.1) |
|  | Private | 165 (41.9) |
| Years of practice | < 5 years | 255 (64.7) |
|  | 5 – 10 years | 89 (22.3) |
|  | >10 years | 50 (13) |
| Attended training on snakebite management | Yes | 102 (25.9) |
|  | No | 292 (74.1) |
| Skills in snake bite management | Self-education (internet or textbooks) | 109 (28.5) |
|  | Learning from senior colleagues | 142 (37) |
|  | Knowledge and skills gained during training | 39 (10.2) |
|  | Don't Know | 94 (24.3) |

**Table 2. The study participants knowledge of snakes and snakebites (n=394).**

|  | **Yes (%)** | **No (%)** | **I don't know (%)** |
| --- | --- | --- | --- |
| **Snake Knowledge** | | | |
| All snakes are poisonous | 36 (9.1) | 321 (81.5) | 37 (9.4) |
| All snakes are carnivorous | 129 (32.7) | 156 (39.6) | 109 (27.7) |
| All snakes are venomous | 94 (23.9) | 255 (64.7) | 45 (11.4) |
| All snake has fangs in front of their mouth | 113 (28.7) | 164 (41.6) | 117 (29.7) |
| Snakes pick sounds using their ears | 71 (18) | 173 (43.9) | 150 (38.1) |
| Snakes are important for farmers | 213 (54.1) | 89 (22.6) | 92 (23.3) |
| Deforestation and urbanisation have increased human-snake interaction | 255 (64.7) | 57 (14.5) | 82 (20.8) |
| **Snakebites Knowledge** | | | |
| Handling a dead snake's head is safe enough | 209 (53) | 85 (21.6) | 100 (25.4) |
| Marks can always be seen or found on the victim after every snake bite? | 161 (40.9) | 195 (49.5) | 38 (9.6) |
| Can a person report at the hospital with symptoms of snake bite without actually being bitten by a snake | 219 (55.6) | 95 (24.1) | 80 (20.3) |
| Sleeping under mosquito nets can prevent snakebites. | 122 (31) | 223 (56.6) | 49 (12.4) |
| A venomous ("poisonous") snake bites always injects venom (poison) into the victim | 172 (53.7) | 151 (38.3) | 71 (18) |
| Signs and symptoms of snake bites are determined by the type of snake responsible for the bite. | 316 (80.2) | 32 (8.1) | 46 (11.7) |
| The signs and symptoms of a snakebite depend on the amount of venom injected by the snake. | 279 (70.8) | 60 (15.2) | 55 (14) |

**Table 3: the study participants knowledge of snakes and snakebites (n =394).**

| **Variable** | **Categories** | **Frequency (%)** |
| --- | --- | --- |
| **Snake Knowledge** | | |
| Animal types of snakes | Mammals | 31 (7.9) |
|  | Birds | 0 (0) |
|  | **Reptiles** | 322 (81.7) |
|  | Not Sure | 41 (10.4) |
| **Snakebites Knowledge** | | |
| Ways to determine snake bitten and venom injected into human | Type of snake | 78 (19.8) |
|  | Signs and symptoms | 209 (53) |
|  | Victim presenting complaints | 55 (14) |
|  | I don't know | 52 (13.2) |
| The common time for snakebite | During the day | 31 (7.9) |
|  | During the night | 274 (69.5) |
|  | Not sure | 65 (22.6) |

**Table 4: The study participants' practice regarding snakebites (n= 394)**

| **Variable** |  | |
| --- | --- | --- |
|  | **Yes (%)** | **No. (%)** |
| A tourniquet prevents the spread of poison in the remaining part of the body | 247 (26.7) | 147 (73.3) |
| Reassurance of the victim should be practice as 1st protocol for prevention | 320 (81.2) | 74 (18.8) |
| Incision at the site of bite helps removing poison | 186 (47.2) | 208 (52.8) |
| If the bite site is leg, elevating it will reduce poison spread | 112 (28.4) | 282 (71.6) |
| Bringing snake to treating physician increases chances of survival by correct identification | 297 (75.4) | 97 (24.6) |

**Table 5: The study participants attitude regarding snakebites (n=394)**

| **Variable** |  | |
| --- | --- | --- |
|  | **Yes (%)** | **No (%)** |
| Residing in cities is a protective factor from snake bite | 297 (75.4) | 97 (24.6) |
| Snakebite is an outcome of revenge inspired by past incidents | 106 (26.9) | 288 (73.1) |

**Table 6: Management of snakebites (n=394)**

| **Variable** | **Response** | **No. (%)** |
| --- | --- | --- |
| Management of snake bites | Very confident | 55 (14) |
|  | Confident | 106 (26.9) |
|  | Fairly confident | 132 (33.5) |
|  | Not confident | 101 (25.6) |
| Have you ever managed, supervised or nursed a snakebite patient before? | Yes | 203 (51.5) |
|  | No | 191 (48.5) |
| Average of snakebites reports to participants health facilities | One person every day | 08 (2) |
|  | 1 to 5 persons a week | 76 (19.3) |
|  | More than five persons a week | 22 (5.6) |
|  | 1 to 5 persons every month | 46 (11.7) |
|  | More than five persons every month | 06 (1.5) |
|  | One person every three months | 11 (2.8) |
|  | More than five persons every three months | 07 (1.8) |
|  | 1 to 5 persons in the year | 28 (7.1) |
|  | More than five persons in the year | 05 (1.3) |
|  | Not Sure | 185 (46.9) |
| The health facility has a protocol for the management of snakebite | Yes | 161 (40.9) |
|  | No | 144 (36.5) |
|  | I don't know | 89 (22.6) |
| The health facility has what it takes to effectively manage snake bites | Yes | 179 (45.4) |
|  | No | 215 (54.6) |

**Table 7: The study participants knowledge of snakebites management (n=394)**

| **Statement** | **Response** | **No. (%)** |
| --- | --- | --- |
| How do you triage (prioritise) someone with a snakebite in your facility? | Emergency | 265 (67.3) |
|  | Urgent | 102 (25.9) |
|  | Not urgent | 07 (1.8) |
|  | I don't know | 20 (5) |
| What do you do when people report to your health facility with snakebite? | Refer immediately | 26 (6.6) |
|  | Give first aid treatments and refer | 166 (42.1) |
|  | Admit and treat | 178 (45.2) |
|  | Call for assistance from another health facility | 24 (6.1) |
| Which of the following tests will you first recommend when someone reports snakebite to determine there was actually an injection of venom into the person (envenoming)? | Full blood count | 182 (46.2) |
|  | Grouping and cross matching | 65 (16.5) |
|  | 20 minutes whole blood count test | 61 (15.5) |
|  | Urinalysis for myoglobinuria | 86 (21.8) |
| Antivenoms are the only antidotes in managing snake bites by venomous snakes. | Yes | 267 (75.4) |
|  | No | 58 (7.1) |
|  | I don't know | 69 (17.5) |
| Antivenoms made anywhere in the world is good for the management of snake bite victims | Yes | 199 (50.5) |
|  | No | 83 (21.1) |
|  | I don't know | 112 (28.4) |
| Should antivenoms be given to all patients bitten by snakes? | Yes | 191 (48.4) |
|  | No | 161 (40.9) |
|  | I don't know | 42 (10.7) |
| When an anti-snake venom is injected into the muscle (intramuscular), it is as effective as when injected into the veins (intravenous) | Yes | 57 (14.5) |
|  | No | 205 (52) |
|  | I don't know | 132 (33.5) |
| Anti-snake venoms remain useful for months or even years after stated expiry dates | Yes | 45 (11.4) |
|  | No | 152 (38.6) |
|  | I don't know | 197 (50) |
| In using anti-snake venoms, it is better to give low doses repeated over several days than high initial doses. | Yes | 132 (33.6) |
|  | No | 118 (29.9) |
|  | I don't know | 144 (36.5) |
