# Supplementary material for: Knowledge and perceptions of snakes, snakebites and their management among health care workers in Sudan
Source: PLoS One. 2024 Sep 20;19(9):e0302698. doi: 10.1371/journal.pone.0302698 (PMC11414984; doi:10.1371/journal.pone.0302698)
Supplement: Attachment — Submitted filename: Rebuttal letter.docx [file pone.0302698.s004.docx]

**Rebuttal letter**

Dear Timothy Omara, PhD
Academic Editor of Plos One Journal

Thank you very much for your informative comments in our manuscript entitled:

**Knowledge and perceptions of snakes, snakebites and their management among health workers in Sudan**.

Hereby the responses for your comments:

1. The title was changed following your recommendation
2. We explain how the sample size was arrived at.
3. The survey will be provided.
4. Numbers were avoided in the starting of sentences.
5. The references were formatted correctly using the same style.
6. Four studies from other African countries were added.
7. The submission benefit from English language proofreading by a proficient speaker.
8. The suggestions were in the attached manuscript file were included.
