# Supplementary material for: Knowledge and perceptions of snakes, snakebites and their management among health care workers in Sudan
Source: PLoS One. 2024 Sep 20;19(9):e0302698. doi: 10.1371/journal.pone.0302698 (PMC11414984; doi:10.1371/journal.pone.0302698)
Supplement: Attachment — Submitted filename: R4 Manuscript KAP snakes (1).docx [file pone.0302698.s006.docx]

**Knowledge and perceptions of snakes, snakebites and their management among health workers in Sudan**

Ali Awadallah Saeed^1,2^, Omer Abdelhamid Gibreel^3^, Ayman B. Mousa^1^, Saeed M. Omer ^4^, Abdallateif Alkhair Omer^5^, Intisar A. M. A. Elalawy ^6^, Ahmed Hassan Fahal^2^

1 Department of Pharmacology and Therapeutics, Faculty of Clinical and Industrial Pharmacy, National University-Sudan.

2 Mycetoma Research Center, University of Khartoum

3 Department of Management Information System, College of Business Administration, Gulf University for Science and Technology, Keuwit

4 Faculty of Medicine and Health Sciences, Gadarif University, Sudan.

5 Faculty of Medicine, Omdurman Islamic University, Sudan.

6 Alrayan College of Health Sciences and Nursing, Pharmacy program, Almadina, Saudi Arabia.

**Corresponding author: Ali Awadallah Saeed**

[**aliawadsaeed@nu.edu.sd**](mailto:aliawadsaeed@nu.edu.sd)

**Abstract:**

**Background:**

Snakebite is a global health crisis recently recognised by the WHO as a neglected tropical disease requiring innovative strategies for mitigation. Adequate knowledge of medical and healthcare providers is lacking.

**Materials and Methods:**

In August 2022, a descriptive cross-sectional survey was conducted involving 394 medical and healthcare providers in snakebite-endemic regions of Sudan. A validated questionnaire was used. It consisted of seven sections addressing the study population demographic characteristics, knowledge of snakes, snakebites, and their management. Data analysis used various statistical tests using Microsoft Excel and the Statistical Package for Social Sciences (SPSS) version 20 (IBM SPSS Inc., Chicago, IL) was done.

**Results:**

Among the 394 participants (44.7% males, 53.3% females), 58.1% demonstrated adequate knowledge of snakes, and 45.3% exhibited adequate knowledge of snakebites. A mere 25.9% received training in snakebite management, with 60.4% possessing adequate knowledge in this domain. Only 14% expressed high confidence in managing snakebites, and 40.9% reported having protocols for snakebite management at their health facilities.

**Conclusion:**

The study highlighted the inadequacy of healthcare providers' knowledge in snakebite-endemic areas. Urgent interventions, such as intensive continuing professional education and training, are essential to address this neglected medical and health problem. Implementation of diverse educational management systems and tools is recommended for comprehensive improvement.

**Introduction**

Snakebite is considered one of the world’s most serious medical problems. The WHO recently declared snakebite envenomation a neglected tropical disease [1]. It called for new strategies to combat it and resources to implement them [2]. There are 5.4 million poisonous snake bites globally, of which around 2.5 million are fatal. Snakebites are accountable for 1,25,000 deaths annually [3]. WHO reported that 81-95% of snake bites occur in the tropical regions of South Asia, Southeast Asia, Sub-Saharan Africa and Latin America [4]. In sub-Saharan Africa, the number of persons treated in health centers for snakebite envenomation is estimated at 315,000 cases per year, with more than 9,000 amputations and 7,000 deaths. Since many victims do not seek medical care, they are invisible to official reports; thus, the number of snake envenomation remains underestimated [5].

In some parts of the African continent, the snakebites' burden is higher than that of trypanosomiasis, leishmaniasis, and onchocerciasis, and its mortality can be higher than malaria [6]. The snakebites' high mortality in Africa is multifactorial, including lack of effective antitoxins, inadequate health care system, and scarcity of rapid access to medical centers [7]. Furthermore, many inhabitants of rural areas believe in traditional healers and do not seek treatment due to low socioeconomic and health education levels, many victims die shortly after the snakebite accident, and that is not reported in the hospital records [8]. In rural regions, where most snakebites occur, the general population's awareness of appropriate behaviour in response to snakebites is poor [9]. A study in Nigeria showed that most patients had attempted at least one potentially deleterious first-aid measure prior to presentation to hospitals [10]. In Ghana, an intervention to improve snakebite medical care ability and adherence to protocols significantly improved care and decreased snakebite mortality [11].

In Sudan, snakebite statistics are lacking despite the high estimated burden of the problem [12]. During the last 70 years, only a few data have been reported on Sudanese venomous snakes, and the Natural History Museum, University of Khartoum, reported some [13,14]. One study reported the presence of 17 medically significant snakes belonging to three major families: Burrowing asps, Elapidae, and Viperidae [15]. These snakes usually become abundant during and after the rainy season, and most snakebite victims are farm workers [16].

This study was set out based on the observed snakebite management, poor outcomes and lack of information on the healthcare provider's knowledge of this serious, deadly medical and health condition.

**Material and methods**

This descriptive cross-sectional survey was conducted in August 2022 using snowball sampling. The study participants were from snakebites envenomation endemic regions of Sudan. It included 394 medical and healthcare providers. A predesigned web-based self-administered questionnaire, validated through a pilot study, was used for data collection. The Sudan Snakebite Research Network developed this questionnaire. The questionnaire was widely distributed online, and all participants gave written informed consent. The questionnaire comprised seven sections covering demographic characteristics, knowledge of snakes (eight questions), knowledge of snakebites (nine questions), practice in dealing with snakebites (five questions), attitude toward affected patients (two questions), and snakebite management (ten questions).

**Data analysis**

The data underwent entry and management using Microsoft Excel and the Statistical Package for Social Sciences (SPSS) version 20 (IBM SPSS Inc., Chicago, IL). Frequency and percentage were computed for qualitative variables, while quantitative variables were characterised by median and interquartile range (IQR). A comprehensive score encompassing knowledge, attitude, and practice was determined and subsequently categorised as adequate or inadequate based on the average score. Percentages were employed to articulate attitudes towards dealing with snakebites. A chi-square test explored associations between adequate knowledge, practice, and selected variables. The analysis of questions involved scoring each correct answer as 1, with "don't know" and incorrect responses receiving 0 points. The result is expressed as percentages. The results were rated adequate and inadequate based on the median.

**Ethical considerations**

Ethical clearance for the study was obtained from the National University Institutional Review Board, and all participants gave written informed consent.

**Results**

The study included 394 medical and healthcare providers, with 176 (44.7%) males and 218 (53.3%) females. Among the participants, 142 (36%) were under 40, while 252 (64%) were 40 or older. The distribution of participants included pharmacists (31%), medical officers (23.9%), registrars (20%), house officers (11.7%), nurses (6.8%), and consultants (6.6%).

Analysis revealed that 255 participants (64.7%) had less than five years of experience in medical and health practice, 89 (22.3%) had experience ranging from five to ten years, and 50 (13%) possessed more than ten years of experience. Regarding training in snakebite management, only 102 participants (25.9%) had received such training. Among them, 37% acquired it from senior colleagues, 28.5% were self-educated, and 10% obtained it through training courses, Table 1.

There were eight questions on snake knowledge, and the results showed that 321 (81.5%) knew the fact that not all snakes are poisonous, 129 (32.7%) stated all snakes are carnivorous, 255 (64.7%) knew not all snakes are venomous, 113 (28.7%) recognised all snakes have fangs in front of their mouth, 213 (54.1%) thought snakes pick sounds using their ears, 213 (54.1%) believed snakes are important for farmers, and 255 (64.7%) believed deforestation and urbanisation have increased human-snake interaction, Table 2). The study showed that 322 participants (81.7%) knew snakes are reptiles, Table 3. Moreover, 229 participants (58.1%) answered snake knowledge questions correctly, while 165 (41.9%) had incorrect answers.

There were nine questions concerning snakebite knowledge, and 85 participants (21.6%) responded correctly to the statement that handling a dead snake's head is not safe enough. Additionally, 195 (49.5%) recognised the fact that marks can always be seen or found on the victim after every snakebite, and 219 (55.6%) believed that a person reported at the hospital with symptoms of snakebite could be given an antitoxin injection without actually being bitten by a snake. Moreover, 122 (31%) believed sleeping under mosquito nets can prevent snakebites. A total of 151 (38.3%) did not think the statement that a venomous ("poisonous") snake always injects venom (poison) into the victim. Furthermore, 316 (80.2%) recognised that the snake type determines the symptoms and signs of snakebites, and 279 (70.8%) thought that the symptoms and signs of the snakebite depend on the amount of venom injected by the snake, Table 2. The obtained data showed that 209 participants (53%) believed the symptoms and signs could determine the snake and venom injected, and 31 (7.9%) felt the snakebite commonly occurs during the day, Table 3. For snakebite knowledge, 179 participants (45.3%) answered the questions correctly, while 215 (54.7%) responded incorrectly.

The study showed that 247 participants (26.7%) believed that a tourniquet prevents the spread of poison in the remaining part of the body, and 320 (81.2%) stated reassurance of the victim should be practiced as the first protocol for prevention. Regarding the belief that an incision at the bite site helps remove the poison, 186 participants (47.2%) held this belief, and 282 (71.6%) thought if the leg is the bite site, its elevation will not reduce poison spread. Moreover, 297 participants (75.4%) believed bringing the snake to the treating physician increases the chances of survival by correctly identifying the snake, Table 4. In this study, 238 participants (60.4%) answered snakebite practice questions correctly, while 156 (39.6%) did not.

Concerning attitudes toward snakebites, residing in cities as a protective factor against snakebites was stated by 297 participants (75.4%), and 106 (26.9%) believed that snakebites result from revenge inspired by past incidents, Table 5.

The study showed that only 55 participants (14%) rated themselves as very confident in managing snakebites, and 203 participants (51.5%) had previously managed a snakebite patient. Regarding management and help provided by participants in snakebite management in 2021, only 20 participants (5.1%) managed more than 100 patients, and 56 (14.2%) managed between 10 and 50 patients. Concerning the availability of snakebite management protocol, 161 (40.9%) stated they have that at their health facilities. Only 179 participants (45.4%) said their health facility has what it takes to manage snakebites effectively, Table 6.

Regarding the management of snakebites, 265 participants (67.3%) considered it an emergency. Additionally, 178 (45.2%) usually admit snakebite patients to hospitals, while 26 participants (6.6%) usually refer patients immediately to other centers.

Sixty-one participants (15.5%) correctly said a 20-minute whole blood count test is the most appropriate test for snakebite management. The statement that antivenoms are the only antidotes in managing snakebites by venomous snakes was agreed upon by 267 participants (75.4%).

Moreover, 199 participants (50.5%) stated antivenoms made anywhere in the world are suitable for managing snakebite victims, and 191 (48.4%) thought that antivenoms should be given to all patients bitten by snakes. Fifty-seven participants (14.5%) believed that the intramuscular injection of an anti-snake venom is as effective as an intravenous one. Furthermore, 197 participants (50%) did not know how long the anti-snake venoms could remain useful after the expiry date. Regarding anti-snake venoms, 132 participants (33.6%) believed it is better to give low doses repeated over several days than high initial doses.

**Discussion:**

Snakebites represent a substantial global medical and health challenge, with the potential for fatal consequences, especially prominent in specific regions. (12) The repercussions extend beyond immediate health concerns, encompassing a significant economic impact on individuals and communities. (17) Those affected by snakebites may encounter enduring disabilities, leading to the loss of livelihoods and heightened poverty, thereby sustaining a cycle of disadvantage. (18) In Sudan, most of the basic information on snakebites is lacking; likewise, the medical and healthcare workers knowledge of this fatal disease, so this study was set out. (19)

In this study, most participants (64.7%) possessed less than five years of practical experience. Only a quarter (25.9%) underwent snakebite management training, which was informal in 90% of them. These findings are serious as the study participants practicing in snakebites endemic regions. These findings align with a report from Kenya Uganda, and Zambia, which showed only 12% of the healthcare workers received formal snakebite management training (20). These findings necessitate designing objectives and continuing professional development training and education for healthcare providers, particularly those working in snakebite-endemic regions. (21)

Managing snakebites affected patients can be challenging, particularly in regions where venomous snakes are prevalent, and healthcare resources may be limited. (21) The difficulties and constraints associated with snakebite management include the limited access to healthcare, lack of antivenom availability, variability in snake venom, difficulty in snake identification, inadequate training of healthcare personnel, financial constraints, cultural beliefs and practices, lack of public awareness, insufficient research and data and climate and environmental factors. (22) These challenges require a comprehensive approach involving improved healthcare infrastructure, better access to antivenom, education and training for healthcare professionals and communities, and increased research to enhance our understanding of snakebite dynamics. (21) All these should be addressed in managing these patients, designing continuing professional development activities and prevention programmes. International collaboration and support are also crucial to developing effective strategies for snakebite management in diverse regions. (23)

In this study, the participants' knowledge of antivenom was inadequate. In Sudan, numerous endemic areas lack access to antivenom, resulting in severe consequences. Multiple factors contribute to the constrained availability of quality antivenom in regions affected by snakebites.(24) The antivenom high production cost renders it unaffordable for many patients. Additionally, numerous distribution challenges stemming from limited infrastructure, poor transportation networks, and inadequate storage facilities further impede antivenom's efficient and timely dissemination to affected areas. (10) Complicating matters is the diverse array of snake species and the variability in venom composition, necessitating the production of different antivenoms tailored to specific snakebites, which is impossible in the country. (25) Medical and healthcare providers should be aware of these issues.

In many parts of the country, cultural values and traditional healing procedures may guide the decision-making process for snakebite victims and families. Some individuals may prefer traditional remedies over modern medical care, resulting in delayed treatment. Hence, healthcare providers need to organise objective health education sessions and events to reduce the burden of late presentation with massive disease and complications. (26)

It is clear from this study that healthcare providers lack snakebite-effective management, and this is in line with many reports from the African continent. [27,28]. Thus, structured and objective training and educational programmes should provided within the medical and health undergraduate and postgraduate curricula.

The data revealed that only 40.9% of our participants have snakebite management protocols, which is in accordance with reports from Uganda and Ghana [21,29]. Snakebite management guidelines are crucial tools that improve healthcare interventions' consistency, quality, and effectiveness. These guidelines significantly reduce the morbidity and mortality associated with snakebites by providing a structured framework for assessment, treatment, and prevention.

Overall, the participants in the study exhibit insufficient knowledge regarding snakes and the management of snakebites, and there is a notable absence of reports on these matters. This underscores the need to conduct surveillance studies to understand their epidemiology better. Such studies are essential for formulating targeted control and prevention measures based on objective insights. (9)

As with other neglected tropical diseases, (30) training healthcare providers on snakebite management using digital facilities is important in improving patient outcomes. It not only addresses accessibility and cost considerations but also promotes interactive, consistent, and up-to-date learning. Such training strategies enhance the preparedness and effectiveness of healthcare professionals in managing snakebite cases.

The primary strength of the present study lies in its substantial sample size, comprising healthcare providers operating in endemic regions. This sample offers valuable insights into the knowledge base of practitioners involved in treating patients affected by snakebites. Nonetheless, conducting more comprehensive investigations is essential to discern details about local health facilities, management guidelines and protocols, patients presentations, and treatment outcomes. Such in-depth studies are crucial for sharing experiences and understanding the intricacies associated with snakebite management in specific localities.

**Conflict of interest**: None

**Source of funding**: Self-funded

**References:**

1. Organisation WH. Available at <http://www.who.int/neglected> diseases/diseases/en/. Accessed on December 2017. Neglected tropical diseases.
2. Chippaux JP. Snakebite envenomation turns again into a neglected tropical disease! J Venom Anim Toxins Incl Trop Dis. 2017; 23:38. https://doi.org/10.1186/s40409-017-0127-6 PMID: 28804495; PubMed Central PMCID: PMCPMC5549382.
3. Chippaux JP. Snake bites: appraisal of the global situation. Bull world health organ. 1998 ;( 5):515-24.
4. Kasturiratne A, Wickremasinghe AR, de Silva N, Gunawardena NK, Pathmeswaran A, Premaratna R, et al. The global burdedn of snake bite: a literature analysis and modeling based on regional estimates of envenoming and deaths. PloS Med. 2008; 5:218.
5. Chippaux JP. Estimate of the burden of snakebites in sub-Saharan Africa: a meta-analytic approach. Toxicon. 2011; 57(4):586–99. https://doi.org/10.1016/j.toxicon.2010.12.022 PMID: 21223975.
6. Khalid H, Azrag RS. Retrospective hospital-based study on snakebite envenomation in Sudan. Trans R Soc Trop Med Hyg. 2021 Sep 3;115(9):992-997. doi: 10.1093/trstmh/trab085. PMID: 34101816; PMCID: PMC8417093.
7. Huda Khalid, Rasha S Azrag, Retrospective hospital-based study on snakebite envenomation in Sudan, Transactions of The Royal Society of Tropical Medicine and Hygiene, Volume 115, Issue 9, September 2021, Pages 992–997, <https://doi.org/10.1093/trstmh/trab085>
8. Chippaux JP. Estimate of the burden of snakebites in sub-Saharan Africa: a meta-analytic approach. Toxicon. 2011; 57(4):586–99. https://doi.org/10.1016/j.toxicon.2010.12.022 PMID: 21223975.
9. Afroz A, Siddiquea BN, Shetty AN, Jackson TNW, Watt AD. Assessing knowledge and awareness regarding snakebite and management of snakebite envenoming in healthcare workers and the general population: A systematic review and meta-analysis. PLoS Negl Trop Dis. 2023 Feb 9;17(2):e0011048. doi: 10.1371/journal.pntd.0011048. PMID: 36757933; PMCID: PMC9910687.
10. Habib AG. Public health aspects of snakebite care in West Africa: perspectives from Nigeria. J Venom Anim Toxins Incl Trop Dis. 2013; 19(1):27. https://doi.org/10.1186/1678-9199-19-27 PMID: 24134780; PubMed Central PMCID: PMCPMC3831819.
11. Visser LE, Kyei-Faried S, Belcher DW. Protocol and monitoring to improve snake bite outcomes in rural Ghana. Trans R Soc Trop Med Hyg. 2004; 98(5):278–83. https://doi.org/10.1016/S0035-9203(03) 00065-8 PMID: 15109550.
12. Kasturiratne A, Wickremasinghe AR, de Silva N et al., The global burden of snakebite: a literature analysis and modeling based on regional estimates of envenoming and deaths, PLoS Med 2008; 5:e218.
13. Khalid H, Azrag RS. Retrospective hospital-based study on snakebite envenomation in Sudan. Trans R Soc Trop Med Hyg. 2021 Sep 3;115(9):992-997. doi: 10.1093/trstmh/trab085. PMID: 34101816; PMCID: PMC8417093.
14. Corkill NL., A guide for the species represented in the collection of natural history museum Khartoum. Natural History Museum Publication no. 3, 1935.
15. Tasoulis T, Isbister GK. A Review and Database of Snake Venom Proteomes. Toxins (Basel). 2017 Sep 18;9(9):290. doi: 10.3390/toxins9090290. PMID: 28927001; PMCID: PMC5618223.
16. Wafula ST, Namakula LN, Ninsiima LR, Ssekamatte NK, Walekhwa AW, Mugume IB, Musoke D. Barriers and opportunities for improving management of snakebites: Perspectives of healthcare workers in Northern Uganda. PLoS One. 2023 Sep 25;18(9):e0291032. doi: 10.1371/journal.pone.0291032. PMID: 37747844; PMCID: PMC10519583.
17. Kruk ME, Gage AD, Arsenault C, Jordan K, Leslie HH, Roder-DeWan S, Adeyi O, Barker P, Daelmans B, Doubova SV, English M, García-Elorrio E, Guanais F, Gureje O, Hirschhorn LR, Jiang L, Kelley E, Lemango ET, Liljestrand J, Malata A, Marchant T, Matsoso MP, Meara JG, Mohanan M, Ndiaye Y, Norheim OF, Reddy KS, Rowe AK, Salomon JA, Thapa G, Twum-Danso NAY, Pate M. High-quality health systems in the Sustainable Development Goals era: time for a revolution. Lancet Glob Health. 2018 Nov;6(11):e1196-e1252. doi: 10.1016/S2214-109X(18)30386-3. Epub 2018 Sep 5. Erratum in: Lancet Glob Health. 2018 Sep 18;: Erratum in: Lancet Glob Health. 2018 Nov;6(11):e1162. Erratum in: Lancet Glob Health. 2021 Aug;9(8):e1067. PMID: 30196093; PMCID: PMC7734391.
18. Francis MF, Vianney SJ, Heitz-Tokpa K, Kreppel K. Risks of snakebite and challenges to seeking and providing treatment for agro-pastoral communities in Tanzania. PLoS One. 2023 Feb 10;18(2):e0280836. doi: 10.1371/journal.pone.0280836. PMID: 36763599; PMCID: PMC9916632.
19. Khalid H, Azrag RS. Retrospective hospital-based study on snakebite envenomation in Sudan. Trans R Soc Trop Med Hyg. 2021 Sep 3;115(9):992-997. doi: 10.1093/trstmh/trab085. PMID: 34101816; PMCID: PMC8417093.
20. Ooms GI, van Oirschot J, Waldmann B, von Bernus S, van den Ham HA, Mantel-Teeuwisse AK, Reed T. The Current State of Snakebite Care in Kenya, Uganda, and Zambia: Healthcare Workers' Perspectives and Knowledge, and Health Facilities' Treatment Capacity. Am J Trop Med Hyg. 2020 Nov 23;104(2):774-782. doi: 10.4269/ajtmh.20-1078. PMID: 33236717; PMCID: PMC7866361.
21. Wafula ST, Namakula LN, Ninsiima LR, Ssekamatte NK, Walekhwa AW, Mugume IB, Musoke D. Barriers and opportunities for improving management of snakebites: Perspectives of healthcare workers in Northern Uganda. PLoS One. 2023 Sep 25;18(9):e0291032. doi: 10.1371/journal.pone.0291032. PMID: 37747844; PMCID: PMC10519583.
22. Patikorn C, Ismail AK, Abidin SAZ, Blanco FB, Blessmann J, Choumlivong K, Comandante JD, Doan UV, Mohamed Ismail Z, Khine YY, Maharani T, Nwe MT, Qamruddin RM, Safferi RS, Santamaria E, Tiglao PJG, Trakulsrichai S, Vasaruchapong T, Chaiyakunapruk N, Taychakhoonavudh S, Othman I. Situation of snakebite, antivenom market and access to antivenoms in ASEAN countries. BMJ Glob Health. 2022 Mar;7(3):e007639. doi: 10.1136/bmjgh-2021-007639. PMID: 35296460; PMCID: PMC8928241.
23. Van Oirschot J, Ooms GI, Waldmann B, Kadam P. Snakebite incidents, prevention and care during COVID-19: Global key-informant experiences. Toxicon X. 2021 Jul 15;9-10:100075. doi: 10.1016/j.toxcx.2021.100075. PMID: 34386765; PMCID: PMC8342778.
24. Potet J, Beran D, Ray N, Alcoba G, Habib AG, Iliyasu G, Waldmann B, Ralph R, Faiz MA, Monteiro WM, de Almeida Gonçalves Sachett J, di Fabio JL, Cortés MLÁ, Brown NI, Williams DJ. Access to antivenoms in the developing world: A multidisciplinary analysis. Toxicon X. 2021 Oct 26;12:100086. doi: 10.1016/j.toxcx.2021.100086. PMID: 34786555; PMCID: PMC8578041.
25. Warrell DA, Gutiérrez JM, Calvete JJ, Williams D. New approaches & technologies of venomics to meet the challenge of human envenoming by snakebites in India. Indian J Med Res. 2013;138(1):38-59. PMID: 24056555; PMCID: PMC3767246.
26. Steinhorst J, Aglanu LM, Ravensbergen SJ, Dari CD, Abass KM, Mireku SO, Adu Poku JK, Enuameh YAK, Blessmann J, Harrison RA, Amuasi JH, Stienstra Y. 'The medicine is not for sale': Practices of traditional healers in snakebite envenoming in Ghana. PLoS Negl Trop Dis. 2021 Apr 16;15(4):e0009298. doi: 10.1371/journal.pntd.0009298. PMID: 33861735; PMCID: PMC8081335.
27. Barnes K, Ngari C, Parkurito S, Wood L, Otundo D, Harrison R, et al. Delays, fears and training needs: Perspectives of health workers on clinical management of snakebite revealed by a qualitative study in Kitui County, Kenya. Toxicon X. 2021; 11:100078.
28. Michael GC, Bala AA, Mohammed M. Snakebite knowledge assessment and training of healthcare professionals in Asia, Africa, and the Middle East: A review. Toxicon X. 2022; 16:100142. Epub 2022/11/29
29. Mabel Worlasi Dzenu, Afaya Agani, Martin Ayanore, Community and health system factors influencing snake envenomation management practices in three districts of Ghana: a qualitative inquiry from health stakeholders and snakebite victims, medRxiv preprint <https://doi.org/10.1101/2023.02.16.23286015>.
30. Bhaumik S, Zwi AB, Norton R, Jagnoor J. How and why snakebite became a global health priority: a policy analysis. BMJ Glob Health. 2023 Aug;8(8):e011923. doi: 10.1136/bmjgh-2023-011923. PMID: 37604596; PMCID: PMC10445399.

**Tables:**

**Knowledge and perceptions of snakes, snakebites and their management among health workers in Sudan.**

**Table 1. Socio-demographic characteristics of the study participants (n=394)**

| **Variables** | **Variable** | **No. (%)** |
| --- | --- | --- |
| Sex | Male | 176 (44.7) |
|  | Female | 218 (53.3) |
| Age | < 40 | 142 (36) |
|  | ≥ 40 | 252 (64) |
| Age | 29.3 ± 8 |  |
| Occupation | Medical officer | 94 (23.9) |
|  | House officer | 46 (11.7) |
|  | Registrar | 79 (20) |
|  | Consultant | 26 (6.6) |
|  | Pharmacist | 122 (31) |
|  | Nurse | 27 (6.8) |
| Working Sector | Governmental | 229 (58.1) |
|  | Private | 165 (41.9) |
| Years of practice | < 5 years | 255 (64.7) |
|  | 5 – 10 years | 89 (22.3) |
|  | >10 years | 50 (13) |
| Attended training on snakebite management | Yes | 102 (25.9) |
|  | No | 292 (74.1) |
| Skills in snake bite management | Self-education (internet or textbooks) | 109 (28.5) |
|  | Learning from senior colleagues | 142 (37) |
|  | Knowledge and skills gained during training | 39 (10.2) |
|  | Don't Know | 94 (24.3) |

**Table 2. The study participants knowledge of snakes and snakebites (n=394).**

|  | **Yes (%)** | **No (%)** | **I don't know (%)** |
| --- | --- | --- | --- |
| **Snake Knowledge** | | | |
| All snakes are venomous | 36 (9.1) | 321 (81.5) | 37 (9.4) |
| All snakes are carnivorous | 129 (32.7) | 156 (39.6) | 109 (27.7) |
| All snakes are venomous | 94 (23.9) | 255 (64.7) | 45 (11.4) |
| All snakes have fangs in front of their mouth | 113 (28.7) | 164 (41.6) | 117 (29.7) |
| Snakes pick sounds using their ears | 71 (18) | 173 (43.9) | 150 (38.1) |
| Snakes are important for farmers | 213 (54.1) | 89 (22.6) | 92 (23.3) |
| Deforestation and urbanisation have increased human-snake interaction | 255 (64.7) | 57 (14.5) | 82 (20.8) |
| **Snakebites Knowledge** | | | |
| Handling a dead snake's head is safe | 209 (53) | 85 (21.6) | 100 (25.4) |
| Marks can always be seen on the victim after every snake bite? | 161 (40.9) | 195 (49.5) | 38 (9.6) |
| Can a person report at the hospital with symptoms of snake bite without actually being bitten by a snake? | 219 (55.6) | 95 (24.1) | 80 (20.3) |
| Sleeping under mosquito nets can prevent snakebites | 122 (31) | 223 (56.6) | 49 (12.4) |
| A venomous snake bite always injects venom into the victim | 172 (53.7) | 151 (38.3) | 71 (18) |
| Signs and symptoms of snake bites are determined by the type of snake responsible for the bite. | 316 (80.2) | 32 (8.1) | 46 (11.7) |
| The signs and symptoms of a snakebite depend on the amount of venom injected by the snake. | 279 (70.8) | 60 (15.2) | 55 (14) |

**Table 3: the study participants knowledge of snakes and snakebites (n =394).**

| **Variable** | **Categories** | **Frequency (%)** |
| --- | --- | --- |
| **Snake Knowledge** | | |
| Animal types of snakes | Mammals | 31 (7.9) |
|  | Birds | 0 (0) |
|  | **Reptiles** | 322 (81.7) |
|  | Not Sure | 41 (10.4) |
| **Snakebite Knowledge** | | |
| Ways to determine snake bitten and venom injected into human | Type of snake | 78 (19.8) |
|  | Signs and symptoms | 209 (53) |
|  | Victim presenting complaints | 55 (14) |
|  | I don't know | 52 (13.2) |
| The common time for snakebite | During the day | 31 (7.9) |
|  | During the night | 274 (69.5) |
|  | Not sure | 65 (22.6) |

**Table 4: The study participants' practice regarding snakebites (n= 394)**

| **Variable** |  | |
| --- | --- | --- |
|  | **Yes (%)** | **No. (%)** |
| A tourniquet prevents the spread of venom in the remaining part of the body | 247 (26.7) | 147 (73.3) |
| Reassurance of the victim should be practiced as the 1st protocol for prevention | 320 (81.2) | 74 (18.8) |
| Incision at the site of bite helps removing venom | 186 (47.2) | 208 (52.8) |
| If the bite site is leg, elevating it will reduce the spread of venom | 112 (28.4) | 282 (71.6) |
| Bringing snake to treating physician increases chances of survival by correct identification | 297 (75.4) | 97 (24.6) |

**Table 5: The study participants attitude regarding snakebites (n=394)**

| **Variable** |  | |
| --- | --- | --- |
|  | **Yes (%)** | **No (%)** |
| Residing in cities is a protective factor from snake bite | 297 (75.4) | 97 (24.6) |
| Snakebite is an outcome of revenge inspired by past incidents | 106 (26.9) | 288 (73.1) |

**Table 6: Management of snakebites (n=394)**

| **Variable** | **Response** | **No. (%)** |
| --- | --- | --- |
| Management of snake bites | Very confident | 55 (14) |
|  | Confident | 106 (26.9) |
|  | Fairly confident | 132 (33.5) |
|  | Not confident | 101 (25.6) |
| Have you ever managed, supervised or nursed a snakebite patient before? | Yes | 203 (51.5) |
|  | No | 191 (48.5) |
| Average of snakebites reports to participants health facilities | One person every day | 08 (2) |
|  | 1 to 5 persons a week | 76 (19.3) |
|  | More than five persons a week | 22 (5.6) |
|  | 1 to 5 persons every month | 46 (11.7) |
|  | More than five persons every month | 06 (1.5) |
|  | One person every three months | 11 (2.8) |
|  | More than five persons every three months | 07 (1.8) |
|  | 1 to 5 persons in the year | 28 (7.1) |
|  | More than five persons in the year | 05 (1.3) |
|  | Not Sure | 185 (46.9) |
| The health facility has a protocol for the management of snakebites | Yes | 161 (40.9) |
|  | No | 144 (36.5) |
|  | I don't know | 89 (22.6) |
| The health facility has what it takes to effectively manage snake bites | Yes | 179 (45.4) |
|  | No | 215 (54.6) |

**Table 7: The study participants knowledge of snakebites management (n=394)**

| **Statement** | **Response** | **No. (%)** |
| --- | --- | --- |
| How do you triage (prioritise) someone with a snakebite in your facility? | Emergency | 265 (67.3) |
|  | Urgent | 102 (25.9) |
|  | Not urgent | 07 (1.8) |
|  | I don't know | 20 (5) |
| What do you do when people report to your health facility with snakebite? | Refer immediately | 26 (6.6) |
|  | Give first aid treatments and refer | 166 (42.1) |
|  | Admit and treat | 178 (45.2) |
|  | Call for assistance from another health facility | 24 (6.1) |
| Which of the following tests will you first recommend when someone reports snakebite to determine there was actually an injection of venom into the person (envenoming)? | Full blood count | 182 (46.2) |
|  | Grouping and cross matching | 65 (16.5) |
|  | 20 minutes whole blood count test | 61 (15.5) |
|  | Urinalysis for myoglobinuria | 86 (21.8) |
| Antivenoms are the only antidotes in managing snake bites by venomous snakes. | Yes | 267 (75.4) |
|  | No | 58 (7.1) |
|  | I don't know | 69 (17.5) |
| Antivenoms made anywhere in the world is good for the management of snake bite victims | Yes | 199 (50.5) |
|  | No | 83 (21.1) |
|  | I don't know | 112 (28.4) |
| Should antivenoms be given to all patients bitten by snakes? | Yes | 191 (48.4) |
|  | No | 161 (40.9) |
|  | I don't know | 42 (10.7) |
| When an anti-snake venom is injected into the muscle (intramuscular), it is as effective as when injected into the veins (intravenous) | Yes | 57 (14.5) |
|  | No | 205 (52) |
|  | I don't know | 132 (33.5) |
| Anti-snake venoms remain useful for months or even years after stated expiry dates | Yes | 45 (11.4) |
|  | No | 152 (38.6) |
|  | I don't know | 197 (50) |
| In using anti-snake venoms, it is better to give low doses repeated over several days than high initial doses. | Yes | 132 (33.6) |
|  | No | 118 (29.9) |
|  | I don't know | 144 (36.5) |
