# Supplementary material for: Knowledge and perceptions of snakes, snakebites and their management among health care workers in Sudan
Source: PLoS One. 2024 Sep 20;19(9):e0302698. doi: 10.1371/journal.pone.0302698 (PMC11414984; doi:10.1371/journal.pone.0302698)
Supplement: Attachment — Submitted filename: Responses to reviewers.docx [file pone.0302698.s007.docx]

**Rebuttal letter**

Dear Academic Editor of Plos One Journal

Thank you very much for your informative comments in our manuscript entitled:

**Knowledge and perceptions of snakes, snakebites and their management among health workers in Sudan**.

**Hereby the responses for reviewers comments:**

Comments to reviewer:

**Reviewer #3: Title: "Assessment of Knowledge and Practices among Medical and Healthcare Providers in Snakebite-Endemic Regions of Sudan"
The paper by Saeed et al is a report on the Knowledge and Practices among medical and healthcare providers in snakebite-endemic regions of Sudan'. Please see below my comments for your consideration. Further comments are provided in the attached document.**

**1. Methodological Shortcomings:**

**- The manuscript's methodology lacks clarity, particularly in its description of the sampling technique. The use of snowball sampling raises questions about the representativeness of the study population. Additionally, the reliance on a self-administered web-based questionnaire introduces a selection bias, excluding individuals with limited access to the internet or those who may be less inclined to participate in online surveys.**

Author comment:

The used of snowball sampling has been extensively used in studies where hard to reach population problem was present, in our case we tried our best to collect samples of our respondents from 4 areas in Sudan known for the high rate of snake bite. We used the method stated by Raifman et al to sample hard to reach population.

In RDS and time-location sampling (TLS), factors that influence inclusion can be estimated and accounted for in an effort to generate representative samples. RDS is particularly equipped to reach the most hidden members of hard-to-reach populations.

(Raifman, S., DeVost, M.A., Digitale, J.C. *et al.* Respondent-Driven Sampling: a Sampling Method for Hard-to-Reach Populations and Beyond. *Curr Epidemiol Rep* **9**, 38–47 (2022). https://doi.org/10.1007/s40471-022-00287-8)

**- The questionnaire's validation process is inadequately reported, leaving the reader uncertain about the tool's reliability and validity. The lack of information regarding the pilot study's outcomes or adjustments made to the questionnaire undermines the study's scientific rigor.**

Author Comment:

After a review of the literature (8, 31), the standardized questionnaire prepared in English, and the draft questionnaire validated by authors for review and comments in order to ensure applicability, suitability, and consistency in our environment, as well as to define the face and content validity.

**2. Data Analysis and Reporting Issues:
- The data analysis section is notably deficient in critical details. The statistical tests employed are vaguely described, and the specific analyses performed using Microsoft Excel and SPSS lack transparency. A more detailed explanation of statistical methods is imperative for readers to assess the validity of the study's findings.
- The presentation of results lacks clarity and coherence. Essential information, such as confidence intervals and measures of variability, is conspicuously absent, hampering the reader's ability to interpret the significance and reliability of the reported results.**

Author comment:

Due to the nature of the study as descriptive exploratory study, none of the inferential statistical method was employed. This is line with numerous similar papers that publish such types of exploratory studies in area of snakes, snakebites and snakebite management.

**3. Incomplete Background and Literature Review:
- The introduction fails to provide a comprehensive background on the existing knowledge and research gaps in the field of snakebite management in Sudan.**

Author comment:

In Sudan, snakebite statistics are lacking despite the high estimated burden of the problem [12]. During the last 70 years, only a few data have been reported on Sudanese venomous snakes, and the Natural History Museum, University of Khartoum, reported some [13,14]. One study reported the presence of 17 medically significant snakes belonging to three major families: Burrowing asps, Elapidae, and Viperidae [15]. These snakes usually become abundant during and after the rainy season, and most snakebite victims are farm workers [16].

**4. Generalization and External Validity:
- The study claims to draw conclusions about the knowledge and practices of healthcare providers in snakebite-endemic regions of Sudan. However, the absence of a clear geographic delineation of the study areas and the use of a convenience sampling method limit the generalizability of the findings to the broader target population**

Author Comment:

The study participants were from snakebites envenomation endemic regions of Sudan (Gaddarif, Sinnar, Khartoum, and Kassala) (figure 1)

**5. Ethical Considerations and Informed Consent:
- The manuscript lacks transparency regarding ethical considerations. While it mentions obtaining ethical clearance, critical details such as the reference number and participant confidentiality measures are missing. This omission raises ethical concerns and undermines the manuscript's scientific integrity.**

Author comment:

- Reference number: NU-REC/11-018/12
- Our study observed all the rights of the participants and conscious of the confidentiality of the information obtained and kept the anonymity of the participants during and after the scheduled period of the study.

Reviewer #2: Please be specific about which group of health workers lack knowledge on snake bite management. Mention the location of study area, mention in which health institution the study was conducted. Mention ratio of health workers to the ratio of health workers participated in the study.

**Author comment:**

In table 1 the healthcare workers participated in the study were there.

**Table 1. Socio-demographic characteristics of the study participants (n=394)**

| **Variables** | **Character** | **No. (%)** |
| --- | --- | --- |
| Occupation | Medical officer | 94 (23.9) |
|  | House officer | 46 (11.7) |
|  | Registrar | 79 (20) |
|  | Consultant | 26 (6.6) |
|  | Pharmacist | 122 (31) |
|  | Nurse | 27 (6.8) |
| Working Sector | Governmental | 229 (58.1) |
|  | Private | 165 (41.9) |

Ali Awadallah Saeed

Corresponding author
