# Supplementary material for: Knowledge and perceptions of snakes, snakebites and their management among health care workers in Sudan
Source: PLoS One. 2024 Sep 20;19(9):e0302698. doi: 10.1371/journal.pone.0302698 (PMC11414984; doi:10.1371/journal.pone.0302698)
Supplement: Attachment — Submitted filename: Rebuttal letter 3.4.2024.docx [file pone.0302698.s009.docx]

**Rebuttal letter**

Dear Academic Editor of Plos One Journal

Thank you very much for your informative comments in our manuscript entitled:

**Knowledge and perceptions of snakes, snakebites and their management among health workers in Sudan**.

Hereby the responses for your comments:

1. The references were formatted correctly using the same style.
2. Four studies from other African countries were added.
3. The suggestions were in the attached manuscript file were included.
4. In Table 1, the average age (29.3 ± 8) deleted, and indicated in the main text.
5. The ages represented in some meaningful ranges.
6. In table 3, the first aspect (Type of snakes) removed.
